# Mothers’ Responses to Children’s Emotions and Children’s Behavior: The Mediating Role of Children’s Emotion Regulation

**DOI:** 10.3390/ejihpe14070129

**Published:** 2024-07-01

**Authors:** Catarina Rolo, Eva Diniz, Alessandra Babore, Tânia Brandão

**Affiliations:** 1 Department of Psychology, Autonomous University of Lisbon, 1169-023 Lisbon, Portugal; catarinarolo13@gmail.com; 2 William James Center for Research, Ispa—Instituto Universitário, 1149-041 Lisbon, Portugal; ediniz@ispa.pt; 3 Department of Psychological, Health and Territorial Sciences, School of Medicine and Health Sciences, University «G. d’Annunzio», 66100 Chieti, Italy; a.babore@unich.it

**Keywords:** mothers’ (un)supportive reactions, children’s emotion regulation, children’s behaviors

## Abstract

While prior research has clearly established links between maternal responses and children’s emotion regulation (ER), the implications of these links for children’s behaviors, especially at school (as reported by their teachers), remain much less explored. This study examined the mediating role of children’s ER in the relationship between maternal reactions to both negative and positive emotions of children and the subsequent behaviors of these children at school. Participants included 56 Portuguese school-aged children (31 boys and 25 girls, aged 6–10 years, mean age = 8.27, SD = 1.27), their mothers (aged 26–55 years, mean age = 38.33, SD = 6.68), and their teachers (n = 7 female teachers) in a multi-informant study. Mothers provided reports on their responses to their children’s emotions and their perceptions of the children’s ER and lability/negativity, while teachers assessed the children’s behavior in the classroom. The results indicated that punitive maternal reactions were associated with greater child lability/negativity, which in turn correlated with increased conduct problems and hyperactivity at school. Conversely, maternal encouragement of expression was linked to reduced lability/negativity, which was associated with fewer emotional symptoms at school. Additionally, maternal problem-focused reactions and guided/empowering responses were associated with reduced child lability/negativity, which in turn correlated with fewer conduct problems and less hyperactivity at school. These findings suggest that maternal responses to children’s emotions can significantly influence children’s behaviors in the classroom via mechanisms involving children’s ER.

## 1. Introduction

### 1.1. Children’s Emotion Regulation

Emotion regulation (ER) is crucial for children’s functioning and is particularly vital during the school years, a critical period for the development of regulatory skills and emotion-related knowledge [1,2,3]. ER has been conceptualized by Thompson [4] as “the extrinsic and intrinsic processes responsible for monitoring, evaluating, and modifying emotional reactions, especially their intensity and temporal features, to achieve one’s goals” (pp. 27–28). In the context of children, ER is commonly assessed using the Emotion Regulation Checklist [5,6], which evaluates two dimensions: emotion regulation and lability/negativity. The former dimension encompasses a child’s emotional skills, including self-awareness and constructive emotional expressiveness, while the latter dimension captures difficulties in ER, such as lack of flexibility, emotional activation, reactivity, anger, dysregulation, or mood lability [6].

Overall, ER is deemed essential for children’s development. Research indicates that difficulties in ER are associated with heightened symptoms of psychopathology and increased aggressive behaviors [7,8,9,10]. Furthermore, children who exhibit poor ER are more prone to engage in risk-taking behaviors, such as substance use [11], and face greater challenges in interpersonal relationships [12] and academic performance [13]. In a recent meta-analytic review of longitudinal studies [14], maladaptive ER was found to be linked to later internalizing/externalizing psychopathology, suggesting that ER should be recognized as a transdiagnostic risk factor for the development of psychopathology in children. Kraft et al. [15], in their meta-analysis, also found that ER plays an important role in children’s psychopathology, with maladaptive ER (e.g., rumination or avoidance) being associated with poor outcomes (e.g., depression, anxiety, aggression, or even addiction). 

According to Morris et al.’s [16] tripartite model, children’s ER is influenced by the family context via three distinct pathways: observation (e.g., modeling and emotion contagion), parenting practices (e.g., emotion coaching and reactions to emotions), and the overall emotional climate of the family (e.g., attachment and parenting styles). Several empirical studies have confirmed these associations. For example, it has been found that maternal difficulties in ER have been associated with children’s emotion lability/negativity [17] or that parents’ orientation to emotion has been associated with better children’s ER [18]. Additionally, poor family functioning has been linked to poor children’s ER [19]. 

In this study, we concentrate on the pathway of parenting practices, specifically by examining parents’ responses to both negative and positive emotions of their children.

### 1.2. Mothers’ Reactions to Emotions and Children’s ER

Children’s ER is likely to be influenced by their parents’ specific reactions to their negative and positive emotions [20]. Theoretical proposals suggest that non-supportive caregiver reactions, such as dismissing, minimizing, or punishing children’s negative emotions, can intensify physiological arousal and, consequently, lead to increased behavioral dysregulation; this may occur as children learn to view their emotions as unacceptable, which may cause them to conceal these emotions [21]. Particularly, punishing negative emotions can signal that certain emotions are not allowed, thereby hindering children’s ability to develop skills to regulate these emotions [20]. On the other hand, supportive reactions from parents can help reduce distress and physiological arousal, aiding in the regulation of emotions in emotionally charged contexts. Such supportive practices enhance children’s ability to identify, label, and express their emotions appropriately, fostering the development of effective ER skills [20,22]. In a longitudinal study [23], maternal warmth and supportive responses to children’s emotions were associated with children’s skills to regulate negative emotions later in adolescence. 

Over recent years, empirical studies have bolstered the notion that parents’ reactions to their children’s emotions significantly affect their children’s ER. For instance, Han and Shaffer [24] observed that mothers’ criticism was positively associated with children’s emotion dysregulation, whereas mothers’ emotional over-involvement had a negative association with children’s emotion dysregulation. Additionally, Fabes et al. [25] reported that children whose parents employed harsh coping strategies in response to negative emotions tended to express their emotions more intensely, leading to social behavioral difficulties. Furthermore, Han et al. [26] discovered that parental supportive reactions were linked to better ER in children, although unsupportive reactions showed no significant associations with children ER. It is important to note that this study was conducted in China, suggesting that cultural differences in ER might play a significant role in interpreting these findings. Other research indicates that children receiving supportive responses to negative emotions generally exhibit better ER skills, whereas those encountering non-supportive responses tend to show poorer ER skills [27,28,29,30]. Additionally, children demonstrate enhanced ER abilities when their mothers are likely to accept their negative emotions and be able to reappraise negative events [31].

While much research has focused on parental responses to children’s negative emotions, the responses to children’s positive emotions have been less frequently examined. For instance, Shewark and Blandon [32], using a sample of children aged 2 to 5 years, found that both mothers’ and fathers’ unsupportive reactions to children’s positive emotions were associated with increased lability/negativity in children. This finding underscores the importance of considering responses to positive emotions as well. Additionally, recent studies have delved into cultural variations in how mothers respond to children’s positive and negative emotions [33,34,35]. These studies generally indicate that mothers’ unsupportive reactions to negative emotions are linked to poorer ER across all cultures. However, the impact of mothers’ supportive reactions to positive emotions varies by culture—being negatively related in Nepal, unrelated in Korea, and positively related in Germany [33]. Furthermore, research suggests that Asian mothers tend to exhibit more unsupportive reactions to both negative and positive emotions compared to mothers in the US and Europe, who typically value self-assertion, independence, and autonomy [34,35]. Nevertheless, there is a significant gap in studies concerning European contexts, particularly in Portugal.

Furthermore, while numerous studies have documented the relationship between parents’ reactions to children’s emotions and the children’s ER, there has been limited exploration of how these interactions influence children’s overall adjustment.

### 1.3. Mothers’ Reactions to Emotions, Children’s ER and Children’s Behavior Problems

Mothers’ reactions to children’s emotions have been shown to impact not only children’s ER but also their behavioral and psychological adjustment over time [36]. Studies have demonstrated that parental punitive or minimizing reactions are associated with lower socioemotional competence [37] and increased externalizing problems [38,39,40]. Conversely, emotion and problem-focused parental responses are linked to fewer internalizing problems [39], enhanced social skills [41], and improved psychological adjustment [35]. In a previous study [42], mothers’ supportive reactions to children’s emotions were linked to fewer behavioral problems when reported by mothers but to more behavioral problems at school when reported by teachers. These findings suggest that while mothers’ supportive reactions may be beneficial at home, they may not have the same positive impact at school. This highlights the need to consider the differential effects of parental supportiveness across the various contexts in which children develop, as well as to consider who is evaluating these children’s behaviors.

While the existing literature clearly demonstrates that parents’ reactions to children’s emotions influence children’s ER, the full emotional and behavioral consequences of this influence have not been comprehensively studied. In one investigation involving school-aged children, researchers found that children’s ER mediated the relationship between parents’ emotion socialization practices (specifically their reactions to children’s negative emotions) and the children’s psychological adjustment, measured by internalizing and externalizing symptoms [43]. However, this study was conducted in China, where cultural norms surrounding emotion socialization may differ significantly from those in other regions, suggesting that cultural context is a critical factor in understanding these dynamics. Additionally, in a longitudinal study, mothers’ supportive responses to children’s emotions at age 5 were associated with improved ER in children at age 10; subsequently, enhanced ER at age 10 led to better adolescent adjustment at age 15, as reported by adolescents and their teachers. Conversely, unsupportive reactions over time were linked to negative outcomes [36]. 

### 1.4. The Present Study

In this study, guided by the models proposed by Eisenberg et al. [20] and Morris et al. [16], we aimed to extend previous research by exploring the mediating role of children’s ER in the relationship between mothers’ reactions to their children’s emotions and the children’s behavior as reported by teachers. This multi-informant study focuses on reports from mothers and teachers, reflecting characteristics of Portuguese society, where mothers are predominantly viewed as the primary caregivers. They are heavily involved in direct care activities and in managing the child’s routines and behaviors [44,45,46], thereby playing a pivotal role in socializing the child’s emotions and behaviors. This influence is crucial for helping children understand and express their emotions [14]. Moreover, in Portugal, which is the country in the European Union where children spend the most daily hours in school (approximately 8 h per day) [47], teachers represent a valuable source of information regarding children’s behavioral adjustments. Thus, this study leverages insights from both mothers and teachers to provide a comprehensive view of children’s emotional and behavioral development.

While previous research has established a connection between mothers’ reactions and children’s ER, these studies often do not assess the outcomes in terms of children’s behaviors, particularly from the perspective of teachers, and typically lack a processual approach. Furthermore, most of the research has concentrated on parents’ responses to children’s negative emotions, leaving the impact of their reactions to positive emotions relatively unexplored. An exception is the study conducted by Shewark and Blandon [32] which investigated mothers’ reactions to children’s positive emotions but was limited to a preschool sample and did not explore the consequences of these reactions on children’s behavior or adjustment. Additionally, while some studies have examined cultural differences in parents’ reactions to both negative and positive emotions of children [33,34,35], they similarly did not assess how these reactions affect children’s overall adjustment. This gap in the literature highlights the need for more comprehensive studies that consider both the positive and negative aspects of emotional responses and their direct effects on children’s behavioral outcomes in various cultural contexts.

Given that most existing studies have focused on preschoolers and considering data suggesting that parents’ emotion socialization practices may have varying impacts depending on the age of the children [48], it is crucial to investigate these dynamics in school-aged children. Studying emotion regulation (ER) and related processes in school-aged children is crucial for several reasons. During this period, children experience significant cognitive development, enabling them to think less egocentrically and become aware of different perspectives [2,49]. This cognitive growth allows them to manage their emotions in socially acceptable ways and develop a more varied repertoire of ER strategies. Additionally, it is during this time that children transition from relying on external aids to using internal cognitive strategies for emotion regulation [50,51]. School provides a favorable context for the development of ER skills, complementing the role of the family [49]. Finally, ER has been identified as an important variable linked to academic engagement and achievement [13,50].

Therefore, we aim to examine the links between mothers’ reactions to children’s emotions and children’s behavior, as well as to explore the potential mediating role of children’s ER on this association. Thus, and based on previous studies, we hypothesize that the following:

**Hypothesis** **1.** *Supportive maternal reactions (e.g., expressive encouragement, emotion-focused reactions, problem-focused reactions) to children’s emotions will promote better ER and reduce lability/negativity, which in turn will lead to fewer behavioral difficulties in the classroom.*

**Hypothesis** **2.** *Unsupportive maternal reactions (e.g., distress reactions, punitive reactions, and minimization reactions) to children’s emotions will make ER difficult and increase lability/negativity, which in turn will lead to more behavioral difficulties in the classroom.*

## 2. Materials and Methods

### 2.1. Participants

The inclusion criteria for participation in this study were being a mother of a child aged 6–10, being able to read and write Portuguese, being over 18 years old, and providing informed consent. 

Participants were recruited from a school in Lisbon, a major city in Portugal. This study included 56 children (31 boys and 25 girls) aged between 6 and 10 (Mage = 8.27; SD = 1.27). Most were Portuguese children (n = 49; 87.5%)—the remaining were from other nationalities. In terms of education, 28% were attending the first year, 18% were attending the second year, 29% were attending the third year, and around 25% were attending the fourth year. The mothers were between 26 and 55 years (Mage = 38.33; SD = 6.68). Most were Portuguese (47; 83.9%). In terms of education, most concluded the secondary school (n = 25; 44.6%) or the higher education (n = 21; 37.5%). About marital status, 35 (62.5%) were married or were living with their partner, 15 (26.8%) were single, and 5 (8.9%) were divorced. One participant refused to answer this question. 

The teachers (n = 7) were all female and were class directors: two from the first year, one from the second year, two from the third year, one from the third and fourth year, and one from the fourth year. The teachers were between 40 and 48 years (M = 45.57; SD = 3.26). 

### 2.2. Measures

#### 2.2.1. Sociodemographic Questionnaire

In the sociodemographic questionnaire, we collected data from children and their mothers regarding sex, age, education, and nationality. Additionally, we gathered information on the marital status of the mothers.

#### 2.2.2. Mothers’ Reactions to Children’s Negative Emotions

The Coping with Children’s Negative Emotions Scale (CCNES) [52,53] was employed to measure mothers’ supportive and unsupportive reactions to their children’s negative emotions. The CCNES comprises 12 scenarios depicting situations in which children might experience negative emotions. Mothers were asked to rate their likely response to each scenario on a scale ranging from 1 (very unlikely) to 7 (very likely). This instrument assesses three types of supportive reactions—expressive encouragement, emotion-focused reactions, and problem-focused reactions—as well as three types of unsupportive reactions—distress reactions, punitive reactions, and minimization reactions. In this study, Cronbach’s alphas were 0.88 for expressive encouragement, 0.85 for emotion-focused reactions, 0.73 for problem-focused reactions, 0.82 for punitive reactions, and 0.71 for minimization reactions. The distress reactions, however, exhibited low internal consistency (0.38) and were consequently excluded from this study.

#### 2.2.3. Mothers’ Reactions to Children’s Positive Emotions

The Coping with Children’s Positive Emotions Scale (CCPES) [54,55] was used to assess mothers’ reactions to their children’s positive emotions. This scale includes five scenarios, comprising a total of 25 items. Mothers were asked to rate how they would likely respond to each scenario on a scale ranging from 1 (very unlikely) to 7 (very likely). The scale measures three types of reactions: negative responses (e.g., minimizing or punishing children when they express positive emotions), guided/empowered responses (i.e., recognizing children’s emotions and guiding the emotion regulation process to help children develop adaptive coping strategies), and guided/external instrumental responses (e.g., using material compensation or rewards, which may limit children’s ability to manage autonomously and adaptively their emotions). In this study, Cronbach’s alphas were 0.74 for negative responses, 0.83 for guided/empowered responses, and 0.65 for external/instrumental responses.

#### 2.2.4. Children’s Emotion Regulation—Reported by Mothers

The Emotion Regulation Checklist (ERC) [5,56] was used to assess children’s ER and lability, as perceived by their mothers. This instrument consists of 24 items, which are rated on a 4-point scale ranging from 1 (almost always) to 4 (never). The ERC is divided into two subscales: one for ER, which includes measures of expression of emotions, empathy, and emotional self-awareness, and one for emotional lability/negativity, which encompasses lack of flexibility, anger dysregulation, and mood lability. In this study, the Cronbach’s alphas for the subscales were 0.60 for ER and 0.71 for lability/negativity.

#### 2.2.5. Children’s Behavior—Reported by Teachers 

The Strengths and Difficulties Questionnaire (SDQ) [56,57] was employed to assess children’s behavior. This tool comprises 25 items, categorized into five dimensions, each containing five items: emotional symptoms, peer problems, conduct problems, hyperactivity, and prosocial behaviors. For this study, we used the teacher-rated version of the SDQ. Items are scored on a 3-point scale ranging from 0 (not true) to 2 (certainly true). Cronbach’s alphas were 0.87 for emotional symptoms, 0.70 for peer problems, 0.76 for conduct problems, 0.86 for hyperactivity, and 0.94 for prosocial behaviors, indicating good reliability across the dimensions.

### 2.3. Procedure

This project received ethical approval from the Ethics Committee of CIP-UAL (Reference: 16-2021). Initially, teachers were contacted to determine their willingness to participate in the study. Upon their agreement, informed consent forms were distributed to the children, who then took them home to their mothers. Children whose mothers signed the consent forms took the study protocol home to be completed by their mothers. Out of 259 informed consent forms distributed, 159 were returned signed. Of the 127 protocols sent home, only 56 were completed and returned for analysis, resulting in a participation rate of 22%. 

### 2.4. Data Analysis

The mediation models were tested using the bootstrapping technique (with 5000 resamples) provided by the PROCESS macro (model 4), developed by Hayes [58]. In these models, parents’ reactions to children’s negative and positive emotions were included as independent variables. Children’s ER, as reported by mothers, served as mediators, while children’s behaviors, as reported by teachers, were treated as dependent variables. To minimize the risk of Type 1 errors, only variables that exhibited significant correlations with either the mediators or the outcomes were included in the models. Indirect effects were considered significant if the 95% confidence interval (CI) did not include zero. Direct, indirect, and total effects are reported. 

## 3. Results

### 3.1. Descriptive and Correlational Analyses

Table 1 presents the means, standard deviations, and correlations among the variables under investigation. We observed a positive association between punitive reactions and lability/negativity while noting a negative association with ER. Expressive encouragement exhibited a negative correlation with lability/negativity and a positive correlation with ER. Problem-focused reactions demonstrated negative associations with both conduct problems and hyperactivity. Similarly, guided-empowered reactions displayed negative correlations with lability/negativity, conduct problems, and hyperactivity.

Furthermore, lability/negativity exhibited positive associations with conduct problems and hyperactivity while showing a negative association with prosocial behaviors.

### 3.2. Mediational Analyses 

**Figure 1:**
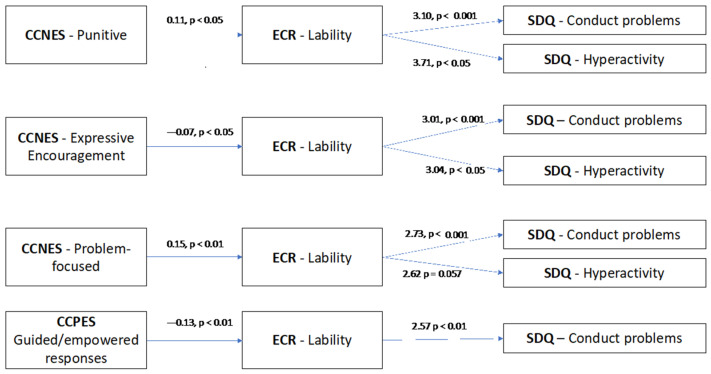
Summary of significant results. Note. CCNES—coping with children’s negative emotions scale; CCPES—coping with children’s positive emotions scale; ECR—emotion regulation checklist; SDQ—strengths and difficulties questionnaire.

Significant associations were found exclusively between punitive reactions, expressive encouragement, problem-focused reactions, and guided-empowered responses with lability/negativity, ER, conduct problems, and hyperactivity. Consequently, our analyses focused solely on models incorporating these variables, resulting in eight distinct models. Figure 1 summarizes the key significant findings derived from these analyses. Complete results are available at Supplemental File (Tables S1–S8). 

#### 3.2.1. Mothers’ Responses to Negative Emotions

We found that punitive reactions were associated with higher lability, which in turn was associated with more conduct problems (indirect effect: 0.33, SE = 0.19; 95% CI [0.049, 0.787]) and hyperactivity (indirect effect: 0.39, SE = 0.23; 95% CI [0.035, 0.940]). Also, expressive encouragement was associated with lower lability, which in turn was associated with fewer conduct problems (indirect effect: −21, SE = 0.11; 95% CI [−0.476, −0.039]) and hyperactivity (indirect effect: −22, SE = 0.13; 95% CI [−0.495, −0.006]). Finally, problem-focused reactions were associated with less lability, which in turn was associated with fewer conduct problems (indirect effect: −41, SE = 0.20; 95% CI [−0.843, −0.086]) and hyperactivity (indirect effect: −40, SE = 0.25; 95% CI [−0.960, −0.005]). 

#### 3.2.2. Mothers’ Responses to Positive Emotions

We found that guided/empowered responses were associated with lower lability, which in turn was associated with fewer conduct problems (indirect effect: −0.34, SE = 0.19; 95% CI [−0.752, −0.055]). The remaining models were not significant. 

## 4. Discussion

In this multi-informant study, we delved into the relationships between mothers’ responses to their children’s positive and negative emotions, the children’s ER (as perceived by their mothers), and the children’s behavioral adjustment in the classroom (as perceived by their teachers). ER stands as a pivotal factor for children’s adaptive socioemotional functioning [3,59], especially in school age [49,51]. Consequently, it is imperative to deepen our understanding of how parental reactions shape children’s ER and the ensuing consequences of this influence on children’s behavior at school. Moreover, previous research has predominantly focused on parental responses to negative emotions, overlooking reactions to positive emotions. Additionally, the emphasis has largely been on preschool-aged children, neglecting the impact on school-aged children. Hence, our study seeks to bridge these gaps in the literature by exploring parental reactions across both positive and negative emotions and by extending the investigation to encompass school-aged children.

Our findings revealed that certain unsupportive maternal reactions to children’s negative and positive emotions are associated with children’s lability/negativity, though not necessarily with their ER, consequently impacting their behavior in the classroom. As hypothesized, punitive reactions exhibited a positive association, whereas expressive encouragement, problem-focused reactions, and guided/empowered reactions (in response to positive emotions) displayed negative associations with children’s lability/negativity [28,32,60,61,62]. These results appear to underscore cultural variations. Punitive reactions typically align with challenges in children’s ER, while supportive responses to both negative and positive emotions appear to be linked to enhanced ER abilities in children, a trend consistent with studies exploring cultural disparities [33,35]. While in certain cultures (e.g., Asian culture), restrictive parenting practices may have a diminished adverse effect on children’s ER [35], in European countries like Portugal, such associations seem to be more adverse.

However, these types of reactions (expressive encouragement, problem-focused reactions, and guided/empowered reactions) did not demonstrate associations with children’s ER, consistent with findings from prior research [30,32,62]. This outcome suggests that mothers’ supportive responses to children’s negative or positive emotions may not directly influence children’s self-awareness or expressiveness. Instead, they appear to be linked to children’s abilities concerning emotional flexibility, activation, and reactivity, or mood lability. Also, it is possible that other variables may moderate or mediate these links (e.g., children’s age, fathers’ emotion socialization; attachment style). For example, in a longitudinal study [63], it was found that while mothers’ unsupportive reactions were associated with more behavior problems at age 5, at age 7, these unsupportive reactions were linked to decreases in these behavioral problems, suggesting that age may play a role on the role played by parent emotion socialization on children’s adjustment. Also, previous studies have shown that both mothers’ and fathers’ responses to children’s emotions are likely to shape children ER [32].

When mothers suppress their child’s negative emotions through punitive behaviors, children may exhibit greater lability/negativity, possibly because they internalize the message that their emotions are invalid and resort to maladaptive strategies for ER [21]. Conversely, when mothers encourage their children to express both negative and positive emotions, offer comfort, or assist them in addressing the cause of distress, they contribute to the development of vital ER skills by reducing lability/negativity. This seems to facilitate effective coping with emotions, as evidenced in previous studies [32,64].

As anticipated, children’s lability/negativity exhibited associations with their behaviors in the classroom, as reported by their teachers. Consistent with prior research [38,40,43], heightened lability/negativity tended to be associated with increased externalizing and problems. In our study, lability/negativity was associated with elevated levels of conduct problems and hyperactivity at school, as reported by teachers. Indeed, emotion dysregulation or difficulties in ER are typically linked with heightened levels of both internalizing and externalizing problems, as well as diminished social competence [18,64]. 

However, it is surprising to note that no significant associations were detected between children’s ER and emotional symptoms or peer problems in our study. Nonetheless, our findings collectively underscore that unsupportive maternal reactions to children’s emotions are likely to be linked with children’s conduct and hyperactivity problems by exerting influence on their ability to regulate emotions. It is important to note that evaluations of children’s adjustment have primarily focused on internalizing and externalizing symptoms, as reported by teachers [17,18,29]. However, other indicators of adjustment also merit attention and further exploration, such as socioemotional competencies, academic performance, and peer relationships. 

While interpreting our findings, it is essential to acknowledge the potential limitations within the scope of our study. Specifically, our research did not comprehensively account for other intervening variables, such as maternal characteristics and mental health. Although our study yielded relevant results concerning the influence of mothers’ reactions to children’s emotions on both children’s ER and behavior, it is important to recognize that the complexity and multicausality inherent in these variables may not have been fully captured. 

### 4.1. Strengths, Limitations, and Future Research

This study has some strengths. This study relied on multiple informants (i.e., mother and teacher-reported data) for assessing children’s ER and children’s classroom behaviors. Also, the present study adds to prior knowledge since it includes mothers’ reactions not only to negative emotions but also to positive emotions. However, some limitations need to be noted. First, our sample is small and only includes mothers. Future studies should use larger samples with mothers and fathers-reported data because it seems that mothers and fathers socialize emotions in a different way [32,61,62]. 

Second, this is a cross-sectional study which limits conclusions regarding causality. Future studies should employ other designs (longitudinal studies) and collect data using other sources (e.g., observation) to better understand causality among study variables. While mediational models were proposed based on theoretical and empirical studies, we recognized that a longitudinal design would be necessary to better ascertain our mediational hypotheses. Also, due to the small sample size, the nature and scope of the analyses were limited, and models had to be tested separately for each subscale. 

Finally, children’s ER was assessed through mothers’ reports, which may lead to some shared bias since they also reported on their own reactions to children’s emotions. Thus, it would be important, in future studies, to consider children’s perspectives regarding both their own ER and their behaviors. 

### 4.2. Clinical Implications

In terms of clinical implications, our findings suggest that it can be important to work with children with maladaptive classroom behaviors by offering them programs that target ER skills to buffer the potential negative effects of unsupportive mother’s reactions. Additionally, it would be important to work also with mothers to promote the development of skills to react to children’s negative and positive emotions with more supportive and adaptive behaviors. 

## Figures and Tables

**Table 1:** Correlations among study variables.

	1.	2.	3.	4.	5.	6.	7.	8.	9.	10.	11.	12.	13.	14.
1. Punitive	-													
2. Minimization	0.789 **	-												
3. Encouragement	−0.097	0.248	-											
4. Emotion focused	0.017	0.196	0.486 **	-										
5. Problem focused	−0.091	0.151	0.707 **	0.722 **	-									
6. Negative	0.617 **	0.333 *	0.013	−0.157	−0.221	-								
7. Guided-empowered	−0.254	−0.052	0.494 **	0.544 **	0.640 **	−0.565 **	-							
8. External-instrumental	0.435 **	0.437 **	0.187	0.544 **	0.311 *	0.182	0.243	-						
9. Lability/negativity	0.311 *	0.026	−0.297 *	−0.184	−0.336 *	0.287	−0.348 **	−0.151	-					
10. Emotion regulation	−0.296 *	−0.235	0.321 *	0.147	0.221	0.032	0.222	−0.016	−0.175	-				
11. Emotional symptoms	−0.186	−0.219	−0.222	−0.139	−0.031	−0.219	0.022	−0.149	−0.141	0.061	-			
12. Peer problems	0.045	−0.045	−0.113	−0.020	0.035	0.021	−0.049	0.048	0.060	0.113	0.582 **	-		
13. Conduct problems	0.118	0.034	−0.124	−0.235	−0.294 *	0.213	−0.337 *	−0.145	0.400 **	−0.009	0.160	0.436 **	-	
14. Hyperactivity	0.075	0.094	−0.180	−0.239	−0.302 *	−0.003	−0.331 *	−0.084	0.347 *	0.072	0.095	0.150	0.714 **	-
15. Prosocial	−0.220	−0.121	0.177	0.012	0.080	0.067	0.049	0.083	−0.285 *	0.187	−0.269 *	−0.651 **	−0.610 **	−0.268

Note. * *p* < 0.05; ** *p* < 0.01.

## Data Availability

The data that support the findings of this study are available on request from the corresponding author. The data are not publicly available due to privacy or ethical restrictions.

## Supplementary Materials

The following supporting information can be downloaded at https://www.mdpi.com/article/10.3390/ejihpe14070129/s1. Tables S1–S8—Direct, total, and indirect effects of the model tested.
