# Supplementary material for: Mothers’ Responses to Children’s Emotions and Children’s Behavior: The Mediating Role of Children’s Emotion Regulation
Source: Eur J Investig Health Psychol Educ. 2024 Jul 1;14(7):1940–52. doi: 10.3390/ejihpe14070129 (PMC11276502; doi:10.3390/ejihpe14070129)
Supplement: Supplementary file 1 [file ejihpe-14-00129-s001.zip › ejihpe-2953584-supplementary.pdf]

Table S1

Total, Direct and Indirect Effects of Punitive Reactions on Conduct Problems through Lability/Negativity and Emotion Regulation

	Coeff	IF	t	p	LLCI	Ulci
Effect of Punitive Reactions on Lability/Negativity	0.1058	0.0440	2.4055	0.0203*	0.0172	0.1944
Effect of Punitive Reactions on Emotion Regulation	-0.1140	0.0469	-2.4327	0.0190*	-0.2084	-0.0196
Effect of Lability/Negativity on Behavior Problems	3.1011	0.8268	3.7506	0.0005**	1.4337	4.7686
Effect of Emotion Regulation on Behavior Problems	0.4053	0.7760	0.5222	0.6042	-1.1597	1.9703
Total Effect of Punitive Reactions on Behavior Problems	0.1918	0.2735	0.7014	0.4867	-0.3590	0.7427
Direct Effect of Punitive Reactions on Behavior Problems	-0.0902	0.2700	-0.3339	0.7401	-0.6347	0.4544
Indirect effects	Effect	Boo SE			Boo 95% LLCI	Boo 95% ULCI
Lability/Negativity	0.3282	0.1863			**0.0494**	**0.7865**
Emotion Regulation	-0.0462	0.1074			-0.2979	0.1478

Note: * $p \leq .05$; ** $p \leq .01$ Coeff=Coefficient; SE=Standard Deviation; LLCI=lower limit of the 95% confidence interval; ULCI= upper limit of the confidence interval 95%; Boo=Bootstrap *results*.

Table S2

Total, Direct and Indirect effects of Punitive Reactions on Hyperactivity through Lability/Negativity and Emotion Regulation

	Coeff	IF	t	p	LLCI	Ulci
Effect of Punitive Reactions on Lability/Negativity	0.1058	0.0440	2.4055	,0203*	,0172	,1944
Effect of Punitive Reactions on Emotion Regulation	-,1140	,0469	-2.4327	,0190*	-,2084	-,0196
Effect of Lability/Negativity on Hyperactivity	3,7059	1,4429	2.5683	,0138*	,7959	6.6159
Effect of Emotion Regulation on Hyperactivity	0.5111	1.3542	0.3774	0.7077	-2.2200	3.2422
Total Effect of Punitive Reactions on Hyperactivity	0.2132	0.4449	0.4793	0.6340	-0.6829	1.1094
Direct Effect of Punitive Reactions on Hyperactivity	-0.1207	0.4712	-0.2561	0.7991	-1.0709	0.8296
Indirect effects	Effect	Boo SE			Boo 95% LLCI	Boo 95% ULCI
Lability/Negativity	0.3922	0.2315			**0.0348**	**0.9401**
Emotion Regulation	-0.0583	0.2024			-0.5149	,3218

Note: * p ≤ . 05; ** p ≤ .01 Coeff=Coefficient; SE=Standard Deviation; LLCI=lower limit of the 95% confidence interval; ULCI= upper limit of the confidence interval 95%; Boo=Bootstrap *results*.

Table S3

Total, Direct, and Indirect effects of Encouragement Reactions on Conduct Problems through Lability/Negativity and Emotion Regulation

	Coeff	IF	t	p	LLCI	Ulci
Effect of Encouragement Reactions on Lability/Negativity	-0.0710	0.0347	-2.0448	0.0462*	-0.1408	-0.0013
Effect of Encouragement Reactions on Emotion Regulation	0.0792	0.0350	2.2649	0.0279*	0.0090	0.1494
Effect of Lability/Negativity on Behavior Problems	3.0111	0.7735	3.8927	0.0003*	1.4558	4.5664
Effect of Emotion Regulation on Behavior Problems	0.0679	0.7687	0.0884	0.9300	-1.4776	1.6135
Total Effect of Encouragement Reactions on Behavior Problems	-0.1878	0.2133	-0.8807	0.3827	-0.6163	0.2406
Direct Effect of Encouragement Reactions on Behavior Problems	0.0206	0.2060	0.1002	0.9206	-0.3935	0.4348

Indirect effects	Effect	Boo SE			Boo 95% LLCI	Boo 95% ULCI
Lability/Negativity	-0.2139	0.1116			**-0.4756**	**-0.0391**
Emotion Regulation	0.0054	0.0740			-0.1666	0.1502

Note: ***** p ≤0.05; ****** p ≤0.01 Coeff=Coefficient; SE=Standard Deviation; LLCI=lower limit of the 95% confidence interval; ULCI= upper limit of the confidence interval 95%; Boo=Bootstrap *results*.

Table S4

Total, Direct, and Indirect effects of Encouragement Reactions on Hyperactivity through Lability/Negativity and Emotion Regulation

	Coeff	IF	t	p	LLCI	Ulci
Effect of Encouragement Reactions on Lability/Negativity	-0.0710	0.0347	-2.0448	0.0462*	-0.1408	-0.0013
Effect of Encouragement Reactions on Emotion Regulation	0.0792	0.0350	2.2649	0.0279*	0.0090	0.1494
Effect of Lability/Negativity on Hyperactivity	3.0403	1.3152	2.3117	0.0251*	0.3959	5.6847
Effect of Emotion Regulation on Hyperactivity	-0.7759	1.3069	-0.5937	0.5555	-3.4037	1.8519
Total Effect of Encouragement Reactions on Hyperactivity	-0.4324	0.3348	-1.2915	0.2025	-1.1049	0.2401
Direct Effect of Encouragement Reactions on Hyperactivity	-0.1551	0.3502	-0.4428	0.6599	-0.8592	0.5490

Indirect effects	Effect	Boo SE			Boo 95% LLCI	Boo 95% ULCI
Lability/Negativity	-0.2159	0.1250			**-0.4947**	**-0.0057**
Emotion Regulation	-0.0614	0.1337			-0.3388	0.2298

Note: * p ≤ . 05; ** p ≤ .01 Coeff=Coefficient; SE=Standard Deviation; LLCI=lower limit of the 95% confidence interval; ULCI= upper limit of the confidence interval 95%; Boo=Bootstrap *results*.

Table S5

Total, Direct, and Indirect effects of Problem-Focused Reactions on Conduct Problems through Lability/Negativity and Emotion Regulation

	Coeff	IF	t	p	LLCI	Ulci
Effect of *Problem-Focused* Reactions on Lability/Negativity	-0.1495	0.0540	-2.7703	0.0078*	-0.2578	-0.0412
Effect of *Problem-Focused* Reactions on Emotion Regulation	0.1184	0.0610	1.9419	0.0577	-0.0040	0.2408
Effect of Lability/Negativity on Behavior Problems	2.7306	0.7834	3.4855	0.0010**	1.1563	4.3050
Effect of Emotion Regulation on Behavior Problems	0.4533	0.6934	0.6537	0.5164	-0.9402	1.8467
Total Effect of *Problem-Focused* Reactions on Behavior Problems	-0.7066	0.3313	-2.1327	0.0378*	-1.3718	-0.0414
Direct Effect of *Problem-Focused* Reactions on Behavior Problems	-0.3521	0.3332	-1.0565	0.2959	-1.0217	0.3176

Indirect effects	Effect	Boo SE			Boo 95% LLCI	Boo 95% ULCI
Lability/Negativity	-0.4082	0.2006			**-0.8432**	**-0.0855**
Emotion Regulation	0.0537	0.0975			-0.1593	0.2511

Note: * p ≤0.05; ** p ≤0.01 Coeff=Coefficient; SE=Standard Deviation; LLCI=lower limit of the 95% confidence interval; ULCI= upper limit of the confidence interval 95%; Boo=Bootstrap *results*.

Table S6

Total, Direct, and Indirect effects of Problem-Focused Reactions in Hyperactivity through Lability/Negativity and Emotion Regulation

	Coeff	IF	t	p	LLCI	Ulci
Effect of *Problem-Focused* Reactions on Lability/Negativity	-0.1495	0.0540	-2.7703	0.0078*	-0.2578	-0.0412
Effect of *Problem-Focused* Reactions on Emotion Regulation	0.1184	0.0610	1.9419	0.0577	-0.0040	0.2408
Effect of Lability/Negativity on Hyperactivity	2.6178	1.3440	1.9477	0.0572	-0.0832	5.3187
Effect of Emotion Regulation on Hyperactivity	0.1238	1.1896	0.1041	0.9175	-2.2667	2.5144
Total Effect of *Problem-Focused* Reactions on Hyperactivity	-1.1856	0.5268	-2.2505	0.0288*	-2.2433	-0.1280
Direct Effect of *Problem-Focused* Reactions on Hyperactivity	-0.8089	0.5717	-1.4151	0.1634	-1.9578	0.3399

Indirect effects	Effect	Boo SE			Boo 95% LLCI	Boo 95% ULCI
Lability/Negativity	-0.3914	0.2456			**-0.9598**	**-0.0050**
Emotion Regulation	0.0147	0.1824			-0.3977	0.4080

Note: * p ≤0.05; ** p ≤0.01 Coeff=Coefficient; SE=Standard Deviation; LLCI=lower limit of the 95% confidence interval; ULCI= upper limit of the confidence interval 95%; Boo=Bootstrap *results*.

Table S7

Total, Direct, and Indirect effects of Guided/empowered Responses on Conduct Problems through Lability/Negativity and Emotion Regulation

	Coeff	IF	t	p	LLCI	Ulci
Effect of Guided/empowered Responses on Lability/Negativity	-0.1312	0.0483	-2.7145	0.0091*	-0.2283	-0.0341
Effect of Guided/empowered Responses on Emotion Regulation	0.0949	0.0547	1.7353	0.0888	-0.0149	0.2047
Effect of Lability/Negativity on Behavior Problems	2.5680	0.7873	3.2616	0.0020*	0.9849	4.1510
Effect of Emotion Regulation on Behavior Problems	0.5454	0.6960	0.7836	0.4371	-0.8540	1.9449
Total Effect of Guided/empowered Responses on Behavior Problems	-0.7402	0.2928	-2.5280	0.0147*	-1.3283	-0.1521
Direct Effect of Guided/empowered Responses on Behavior Problems	-0.4549	0.2954	-1.5400	0.1301	-1.0489	0.1390
Indirect effects	Effect	Boo SE			Boo 95% LLCI	Boo 95% ULCI
Lability/Negativity	-0.3370	0.1855			**-0.7523**	**-0.0554**
Emotion Regulation	0.0518	0.0727			-0.1046	0.1997

Note: * p ≤0.05; ** p ≤0.01 Coeff=Coefficient; SE=Standard Deviation; LLCI=lower limit of the 95% confidence interval; ULCI= upper limit of the confidence interval 95%; Boo=Bootstrap *results*.

Table S8

Total, Direct, and Indirect effects of Guided/empowered Responses on Hyperactivity through Lability/Negativity and Emotion Regulation

	Coeff	IF	t	p	LLCI	Ulci
Effect of Guided/empowered Responses on Lability/Negativity	-0.1312	0.0483	-2.7145	0.0091*	-0.2283	-0.0341
Effect of Guided/empowered Responses on Emotion Regulation	0.0949	0.0547	1.7353	0.0888	-0.0149	0.2047
Effect of Lability/Negativity on Hyperactivity	2.4521	1.3570	1.8070	0.0770	-0.2763	5.1805
Effect of Emotion Regulation on Hyperactivity	0.1984	1.1996	0.1654	0.8693	-2.2136	2.6104
Total Effect of Guided/empowered Responses on Hyperactivity	-1.1633	0.4697	-2.4765	0.0167*	-2.1069	-0.2198
Direct Effect of Guided/empowered Responses on Hyperactivity	-0.8604	0.5092	-1.6898	0.0976	-1.8841	0.1634

Indirect effects	Effect	Boo SE			Boo 95% LLCI	Boo 95% ULCI
Lability/Negativity	-0.3218	0.2411			-0.9093	0.0012
Emotion Regulation	0.0188	0.1391			-0.2790	0.3052

Note: * p ≤0.05; ** p ≤0.01 Coeff=Coefficient; SE=Standard Deviation; LLCI=lower limit of the 95% confidence interval; ULCI= upper limit of the confidence interval 95%; Boo=Bootstrap *results*.
